# Circulating microRNA-122 in HCV cirrhotic patients with high frequency of genotype 3

**DOI:** 10.1371/journal.pone.0268526

**Published:** 2022-05-26

**Authors:** Amin Ullah, Xiaojie Yu, Margarete Odenthal, Sonja Meemboor, Bashir Ahmad, Irshad ur Rehman, Jamshaid Ahmad, Qurban Ali, Tariq Nadeem

**Affiliations:** 1 Centre of Biotechnology & Microbiology (COBAM), University of Peshawar, Peshawar, Pakistan; 2 Centre for Molecular Medicine Cologne (CMMC), University of Cologne, Cologne, Germany; 3 Institute of Pathology, University Hospital of Cologne, Cologne, Germany; 4 Institute of Molecular Biology and Biotechnology, University of Lahore, Lahore, Pakistan; 5 Department of Plant Breeding and Genetics, Faculty of Agriculture, University of the Punjab, Lahore, Pakistan; 6 National Center of Excellence in Molecular Biology, University of the Punjab, Lahore, Pakistan; Texas A&M University, UNITED STATES

## Abstract

MicroRNA-122 (miR-122) is a liver abundant microRNA that is released upon liver injury. In the present study, we investigated the circulating miR-122 profiles in a Pakistani patients´ cohort with HCV chronic liver disease that was mainly based on HCV genotype 3 infections. From 222 patients with chronic HCV liver disease, classified as mild, moderate, or severe, serum samples were collected. Cell-free RNA was isolated and used for miR-122 quantification by qPCR. More than 60% of 222 patients were infected with HCV genotype 3. ALT values and HCV viral load showed no correlation with the HCV genotype. Circulating miR-122 levels were significantly upregulated in patients with cirrhosis. Notably, HCV patients with mild cirrhosis showed the most marked increase in serum miR-122 levels (p = 0.0001). Furthermore, we proved a positive correlation (r = 0.46) of miR-122 with the ALT values in patients with mild cirrhosis. Importantly, our data of increased miR-122 levels in serum samples obtained from a patient cohort with a high prevalence of chronic genotype 3 HCV infection confirmed the previous findings collected from cohorts with a high prevalence of genotype 1. Therefore, we suggest that miR-122 increase after HCV infection does not depend on the HCV genotype. In conclusion, our findings confirm that serum miR-122 levels are significantly upregulated in the HCV cirrhotic patients serving in particular as a biomarker for the non-advanced stages of cirrhosis, independently of the HCV genotype.

## Background

Hepatitis C virus (HCV) is in the hepacivirus genus of the Flaviviridae family, with 9.6 kb single-stranded RNA having codons for 3010 amino acids. HCV infection progresses with a high frequency to cirrhosis, and finally hepatocellular carcinoma (HCC). Liver cirrhosis manifests in structural deterioration of the liver tissues due to necrosis and the mortality rate tenth highest in Pakistan and worldwide [1, 2]. Around 170 to 200 million patients suffer worldwide of HCV infection of which 2.7 to 3.9 million are in the US [3]. According to the World Health Organization (WHO), about 1.412.000 deaths (796.000 due to cirrhosis and 616.000 due to liver carcinoma) are caused by HCV [4–6]. Until now, there is no vaccine against HCV [7].

Nearly 98% of the untranslated human genome is transcribed into non-coding RNAs (ncRNAs). These ncRNAs are long, short-chain, and do not translate into proteins. The 21–23 nucleotides long ncRNAs and short-chain are called microRNAs (miRNAs). miRNAs are the least mutating and most well-studied type of ncRNAs [8]. These miRNAs are present in almost all organisms, controlling gene expression of approximately 30% genes [9], including cancer-related integrin genes [10, 11]. Due to this gene regulation ability, the miRNAs play a significant role in cell differentiation, programmed cell death, embryonic development, and cancer control, etc. [12–14]. These miRNAs can base pairs within the organism as well as with the RNAs of the invading pathogens [15, 16]. MicroRNA-122 (miR-122) is known for complementary base pairing with the 5-UTR region of the HCV RNA, promoting viral RNA replication in hepatocytes [17]. MicroRNA-122 is highly abundant in the liver, affecting lipid metabolism, gene expression, and insulin resistance [18]. Since miR-122 triggers HCV replication by different mechanisms such as enhancing HCV RNA translation and protecting HCV genome RNA degradation, its inhibition is a promising therapeutic approach in recent HCV treatment concepts [19].

Presently, there is only limited information about miR-122 profiles in HCV cohorts with the high frequency of genotype 3. HCV genotype 3 infections are prominently occurring in the Asian population [20]. Patients with chronic HCV genotype 3 infections suffer from fast progression of fibrogenesis and a high incidence of hepatocellular carcinoma (HCC) [21]. Furthermore, HCV genotype 3 frequently leads to a change in lipid metabolism [22] and severe steatosis [23].

## Patients and methods

### Ethical approval

The study was approved by the Ethical Committee, Center of Biotechnology & Microbiology (COBAM), University of Peshawar, Pakistan. All experiments were performed in accordance with relevant named guidelines and regulations, and written informed consent was obtained from all the healthy controls and recruited patients, whose data containing demographic, clinical characteristics, and estimated infection time and miR-122 levels were used.

### Criteria

Patients with chronic liver disease, who were positive by PCR targeting the 5′ UTR of HCV RNA, were included in our study. Co-infected patients i.e. HAV or HBV or HDV, quasispecies, or patients with low viral load, or with no other required information were not included in current research work.

### Patients

Samples were collected from the tertiary care hospital of Peshawar (Pakistan) along with demographic data of the patients (Table 1) living in the region of Khyber Pakhtunkhwa, Pakistan. Child Turcotte Pugh score (CTP) was used for cirrhosis staging where the Score system with 5–6 was referred to as Child Class A (mild), 7–9 as Child Class B (moderate) and 10–15 as Child Class C (severe) of liver disease. Identification of HCV 5′ UTR and genotyping were performed as previously described by the procedure of Ullah et al. [1].

**Table 1:** Demographic characteristics of 222 HCV cohort and 52 healthy control.

Parameters	Patients
**Gende**r	
Male	136 (61%)
Female	86 (39%)
**Age**	
mean± standard deviation(SD) years	49.03± 12.65
mode	50
Healthy Control	
Male	28 (53.84)
Female	24 (26.16)
Age	
mean± standard deviation(SD) years	40± 12.20
ALT level- mean± SD	45± 3.6
**Cirrhosis**	
Mild (n)	37
mean± SD years	50.56± 13.19
mode	50
Moderate (n)	87
mean± SD years	47.18± 12.25
Mode	55
Gross (n)	101
mean± SD years	48.0± 11.94
mode	50
**AST**	
mean± standard deviation	121.29± 28.01
Mode	122
Significant differences	p< 0.0001
**ALT**	
mean± SD	121.40± 29.03
mode	139
mild(mean± SD)	90.05.± 17.11
moderate(mean± SD)	112.57.± 23.59
gross(mean± SD)	140.24.± 22.40
significant differences	p< 0.0001
AFP	
mean± standard deviation	71.05± 57.09
mode	40
mild(mean± SD)	32.59.± 7.77
moderate(mean± SD)	35.51.± 5.58
gross(mean± SD)	114.54.± 60.37
significant differences	p< 0.0001
**Bilirubin mg/dl**	
mean± standard deviation	1.3±0.6
**Size of spleen**	
Enlarge (n)	117(52.70%)
Normal (n)	105(47.29%)
**Blood pressure**	
High (n)	51(22.97%)
Low(n)	91(40.99%)
Normal(n)	80(36.03%)
**Ascites**	
Less(n)	26(11.71%)
Moderate(n)	90(40.54%)
High(n)	106(47.74%)
**Respiratory system**	
Normal(n)	206(92.79%)
Abnormal (n)	16(7.20%)
**Cardiovascular**	
Normal	108(48.64%)
Low	16(7.20%)
High	101(45.49%)

Abbreviations: ALT: alaninamino transferase, AST: aspartate aminotransferase, AFP: alpha fetoprotein, SD: standard deviation.

### RNA isolation

Blood samples of HCV cirrhotic patients were collected, centrifuged at 3.000 rpm for 10 minutes at 4°C, and stored at -80°C. RNA was extracted from the serum samples using the manufacturing protocol of Qiagen Kit (Qiagen, Hilden, Germany. Cat No./ID: 217204). SV40-miRNA (Qiagen) was transferred to serum samples (2 pmol/200 ml) before the RNA extraction method for later normalization of miR-122 levels.

### cDNA and real-time PCR

MicroRNA was analyzed by a two-step real-time PCR and complementary DNA was prepared by the miScript-Reverse Transcription Kit (Qiagen kit. Cat No./ID: 218160) and the miR-SYBR Green PCR Kit (Qiagen, Hilden, Germany. Cat No./ID: 218073). The miR-122 and SV-40 primers were used for cDNA synthesis and cycling protocol followed was; 37°C / 60 min, 95°C / 10 min and 22°C / ꭃ.

Thermo cycling conditions of the RT-PCR were as follows: initial denaturation 95°C/3 min, following 49 cycles of PCR template denaturation 94°C / 30 sec, annealing 55°C / 45 sec and extension 70°C / 45 sec. The melting curve analysis was done at 50–95°C / 0.5 sec. All the steps were done in triplicate and according to the supplier’s guidelines. Spike-in SV40-miRNA (Qiagen, Hilden, Germany. Cat No./ID: 331535) was used for normalization of extracellular miR-122 levels.

### Statistical analysis

Statistical analysis was performed in IBM SPSS software 25.0 and GraphPad Prism 5. The mean, standard deviation, ranges different other variables are reported in Table 1. A one-way ANOVA test was performed for the statistical significance between the groups. Student test (t-test) was performed for the significance between two variables. The significance of ROC, AUC curve, sensitivity, specificity, positive and negative values were studied for diagnostic precision of miR-122. Correlation (Pearson Correlation = r) was performed for two variables that are linearly correlated. The values which are less than 0.05 were considered to be statistically significant (p).

## Results

### Characteristics of patients

**Fig 1:**
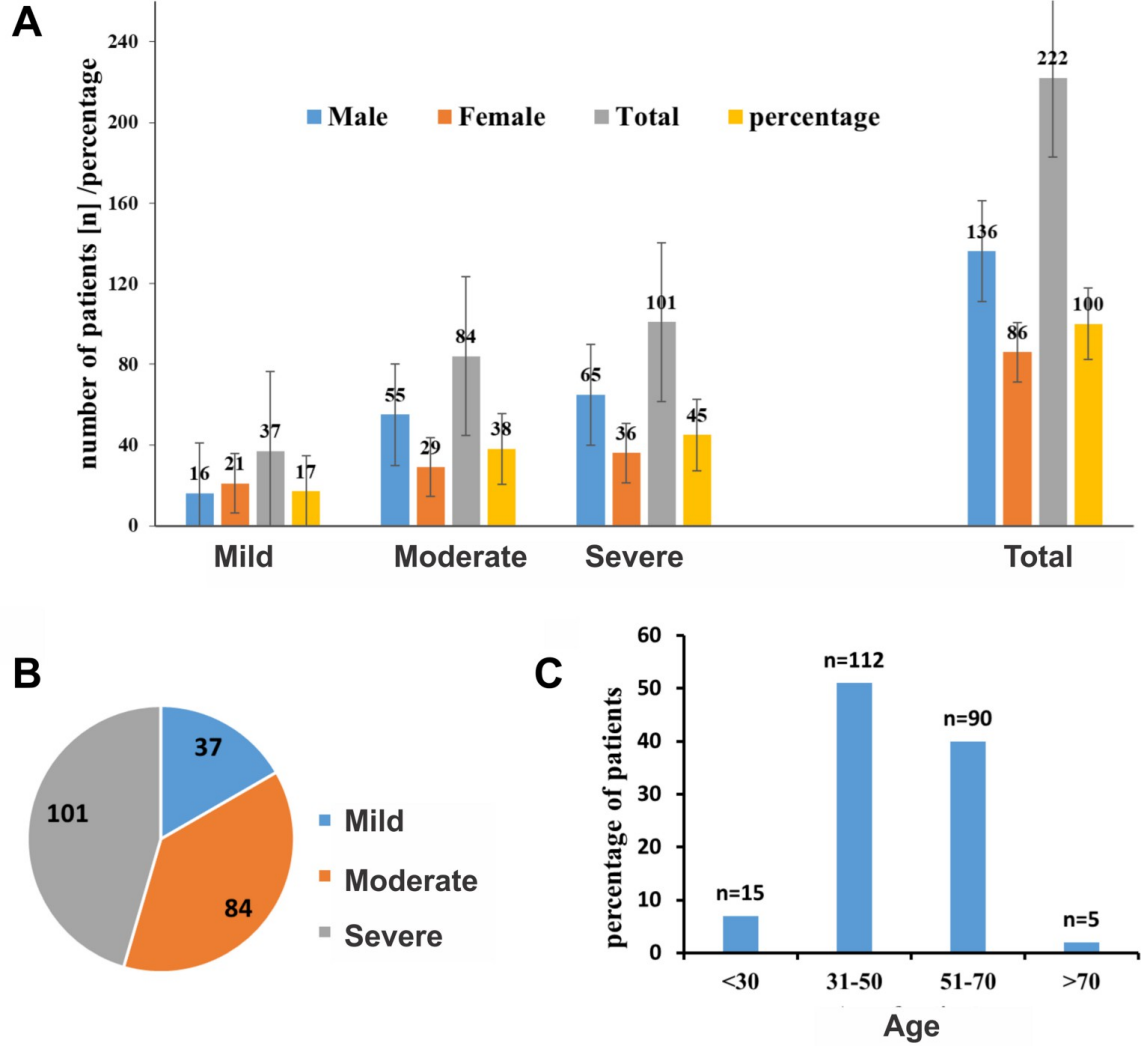
**A:** Distribution of chronically HCV infected patients suffering from mild, moderate, or severe cirrhosis. **B:** Distribution of the HCV infected patients in respect to their cirrhosis stage (mild, moderate, and severe). **C:** Age distribution of HCV cirrhotic patients.

**Fig 2:**
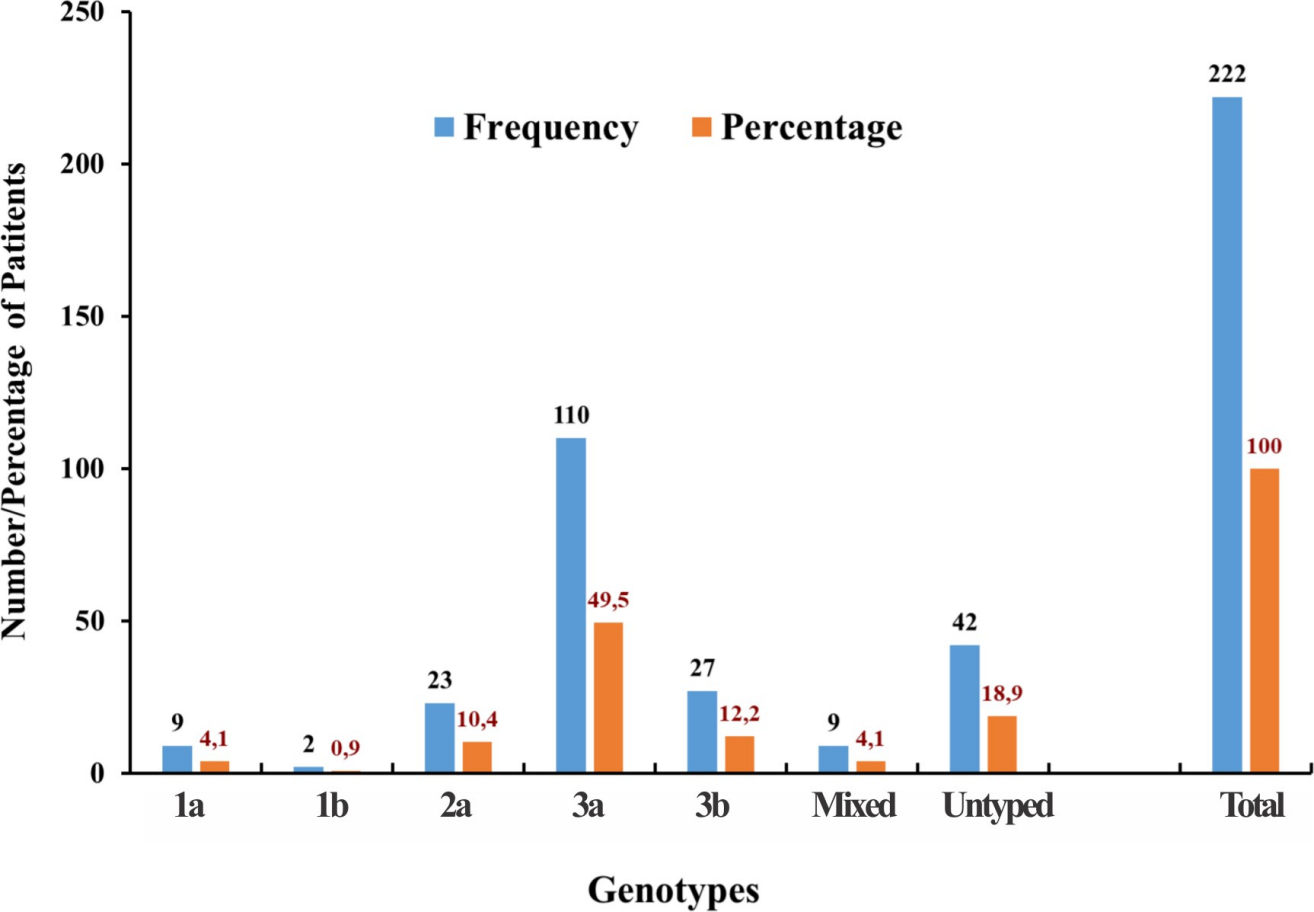
Distribution of HCV genotypes (1b, 2a, 3a, 3b, mixed and untyped) in the patients with chronic HCV hepatitis.

The HCV cirrhotic patients, who agreed to the study, were admitted to the tertiary hospitals Peshawar from April 2016 to October 2018. The demographic summary and clinical information of the patients are shown in Table 1. The HCV cirrhotic patients were categorized into three groups of mild, moderate, and severe cirrhosis. The frequency of patients with severe cirrhosis (n = 101) was higher than those suffering from moderate (n = 87) and mild (n = 37) cirrhosis (Fig 1A and 1B). The mean age of the patients was 49 ± 12 years. Patients, aged 31 to 50 years, reported the highest frequency with severe cirrhosis (Fig 1C). Furthermore, there were significant differences of the alanine transaminase (ALT) and alpha-fetoprotein (AFP) values between the patient groups with no cirrhosis or mild, moderate, and severe cirrhosis, respectively (p< 0.0001 c.f. Table 1). Genotype 3 was the most frequent in the studied cohort (Fig 2) because the samples were collected from patients living in the region of Khyber Pakhtunkhwa in Pakistan, where the HCV genotype 3 is the most frequent HCV genotype of chronic HCV hepatitis (Fig 2). Since in our cohort were only a limited number of patients who suffered from a genotype 1 infection, we compared the liver enzymes and miR-122 levels between HCV genotype 3 carriers and non-HCV genotype carriers.

**Fig 3:**
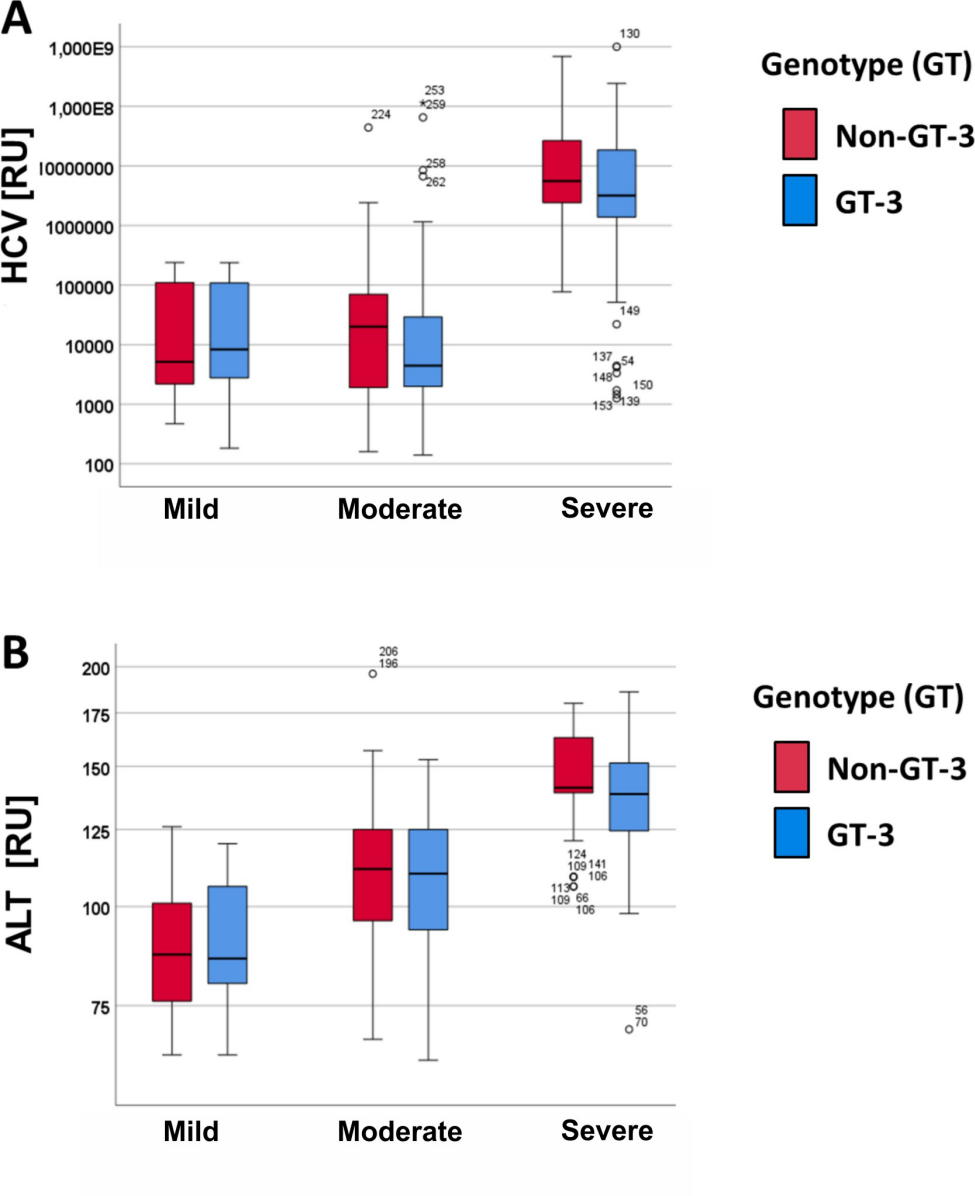
Comparison of non-genotype 3 vs. genotype 3 in patients with mild, moderate and severe cirrhosis. **A:** HCV, **B:** ALT.

There was no difference observed in the viral load of HCV genotype 3 and non-genotype 3 infections (Fig 3A). Furthermore, the ALT values which are increasing with the progression of chronic HCV hepatitis, showing the highest levels in patients with severe cirrhosis, were equally distributed in patients infected with the HCV genotype 3 versus patients with non-genotype 3 HCV genotypes (Fig 3B). Furthermore, there was no difference in the ALT and HCV values between the genotype 3 and non-genotype 3 HCV infected female and male patients (data not shown).

### Expression of miR-122 in HCV cirrhotic patients

**Fig 4:**
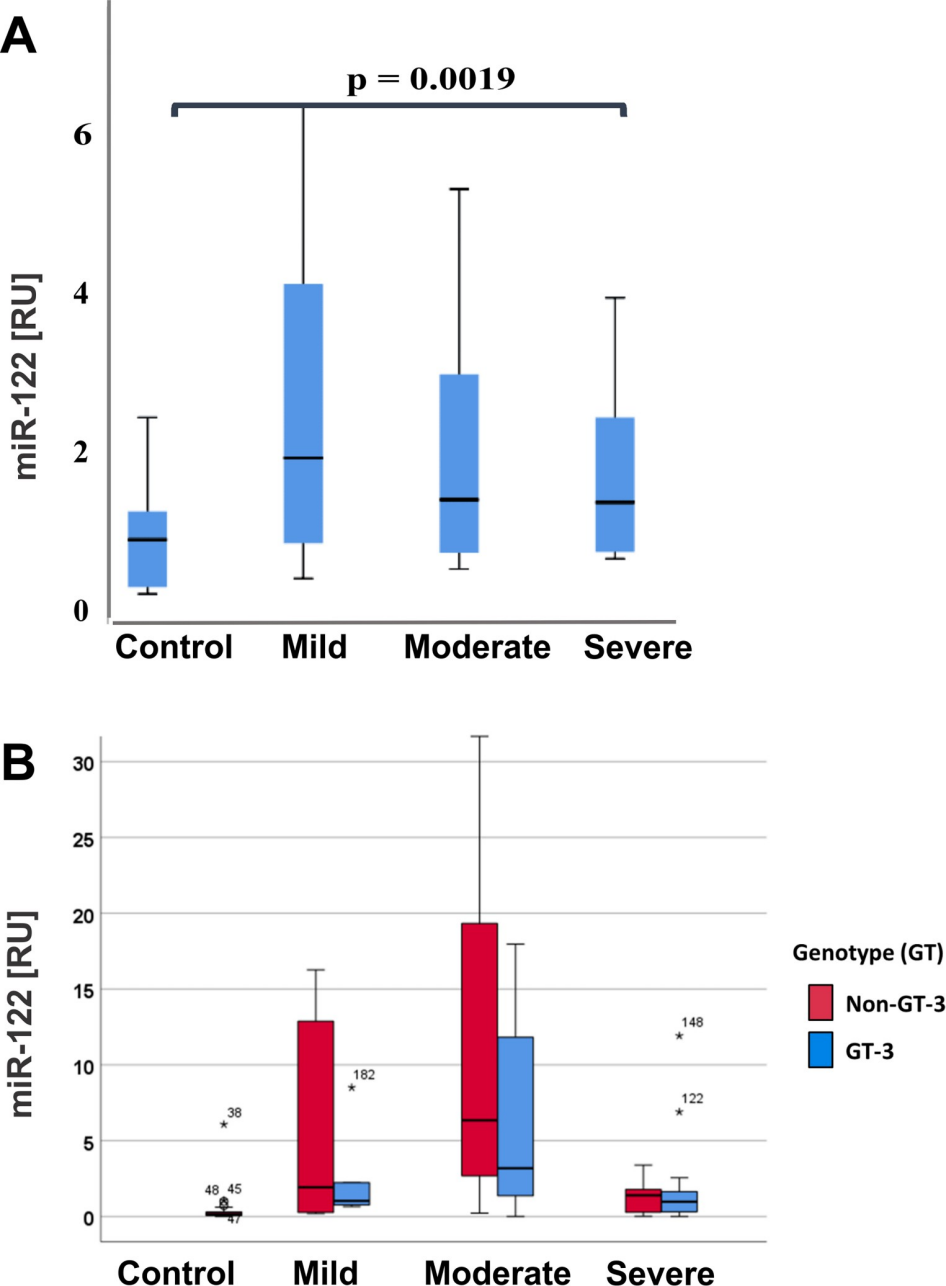
Expression levels of miR-122 in HCV cirrhotic patients. **A:** shows the miR-122 levels in the control group and patients with mild, moderate, and severe cirrhosis. The miR-122 levels were significantly upregulated in the HCV group with mild, moderate, and severe cirrhosis. The data of ΔCq of the normalized expression miR-122 values are presented as Whisker box plots. **B:** Comparison of expression levels of miR-122 in Genotype 3 (in blue) vs. Non-Genotype 3 (in red).

MicroRNA-122 expression levels were quantified by qRT-PCR and the expression of the control group was compared with the mild, moderate, and severe patients (Fig 4A). In evaluation of hepatic miR-122, it was observed that circulating miR-122 was significantly upregulated in the HCV cirrhotic patients compared with control (ANOVA p = 0.0019) as shown in Fig 4 and S1A Fig. The comparison of the miR-122 levels in the control patients with the serum miR-122 values of patients with mild, moderate, and severe cirrhosis showed a significant upregulation (_control vs mild_ = 0.0001, _control vs moderate_ = 0.0032, _control vs severe_ = 0.0001) (S1A–S1C Fig, S1 Table). The increased miR-122 profiles during development of cirrhosis were equally found in HCV infected females and males. The comparison of the miR-122 levels with the ALT values revealed a positive correlation but there were no significant correlation with AFP values. The significant difference was also shown by Tukey’s Multiple Comparison Test as well as Bartlett’s test which was performed to show significant equal variances in the HCV cirrhotic groups (S1 Table).

**Fig 5:**
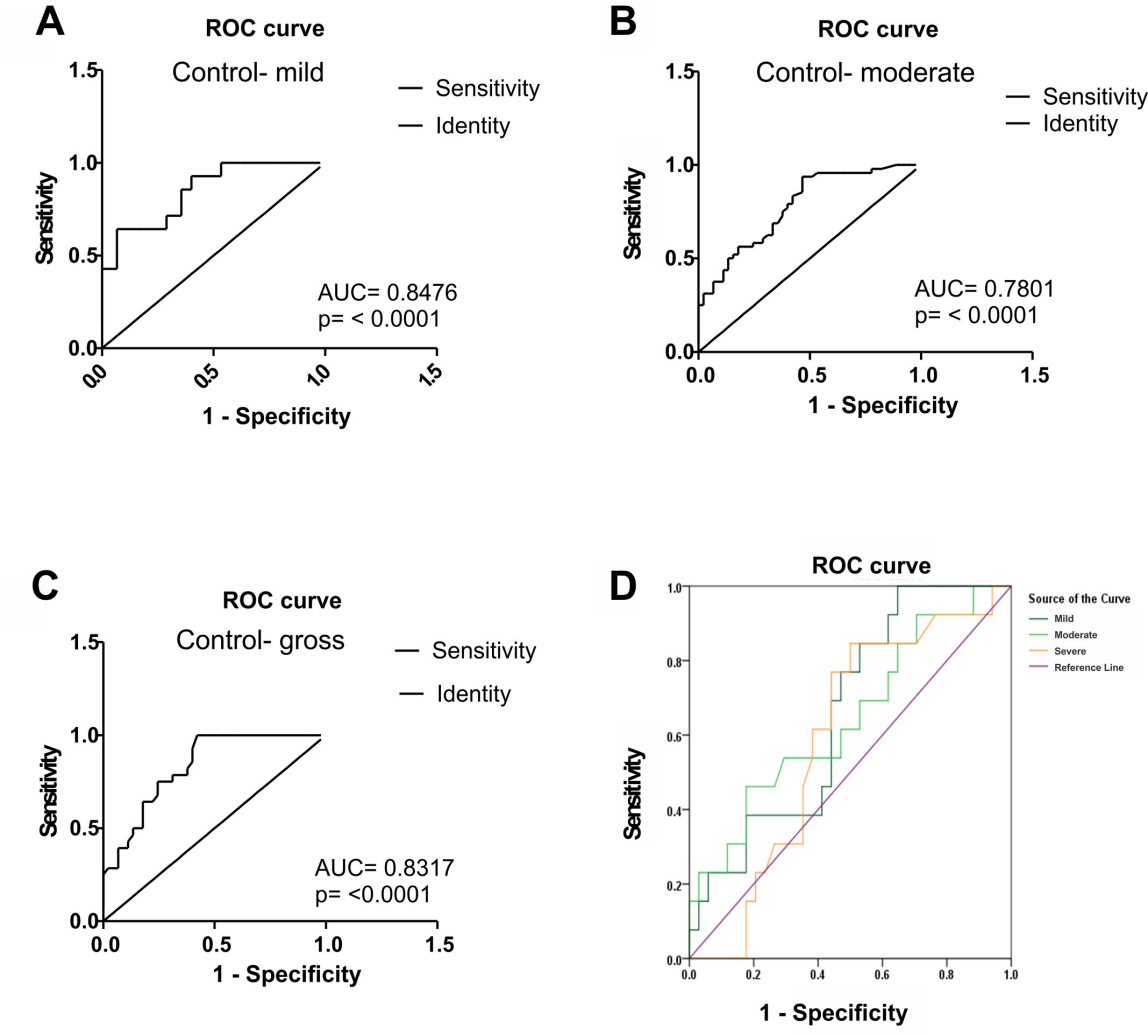
Receiver Operating Characteristic (ROC) curve of serum miR-122 in the HCV patients. ROC curves have been drawn and AUC curves were used to assess and examine the diagnostic ability of miR-122. **A-C:** The miR-122 healthy control was compared to HCV cirrhotic patients (mild, moderate, and severe group). **D:** shows the combined ROC curve of the HCV patients’ groups.

Next, we performed the analysis of receiver operating characteristics (ROC). The area under the curve (AUC) revealed the potential of miR-122 to discriminate between healthy blood donors and patients with chronic HCV liver disease (Fig 5, S1 Table). Though miR-122 levels efficiently indicate moderate and severe cirrhosis, the sensitivity of increased miR-122 levels was highest to prove in mild HCV cirrhosis (Fig 5A–5C). Also, the ROC analysis showed that there was no difference between non-genotype 3 and genotype 3 based HCV hepatitis (data not shown). Fig 5D indicates the collective significance of ROC curves.

**Fig 6:**
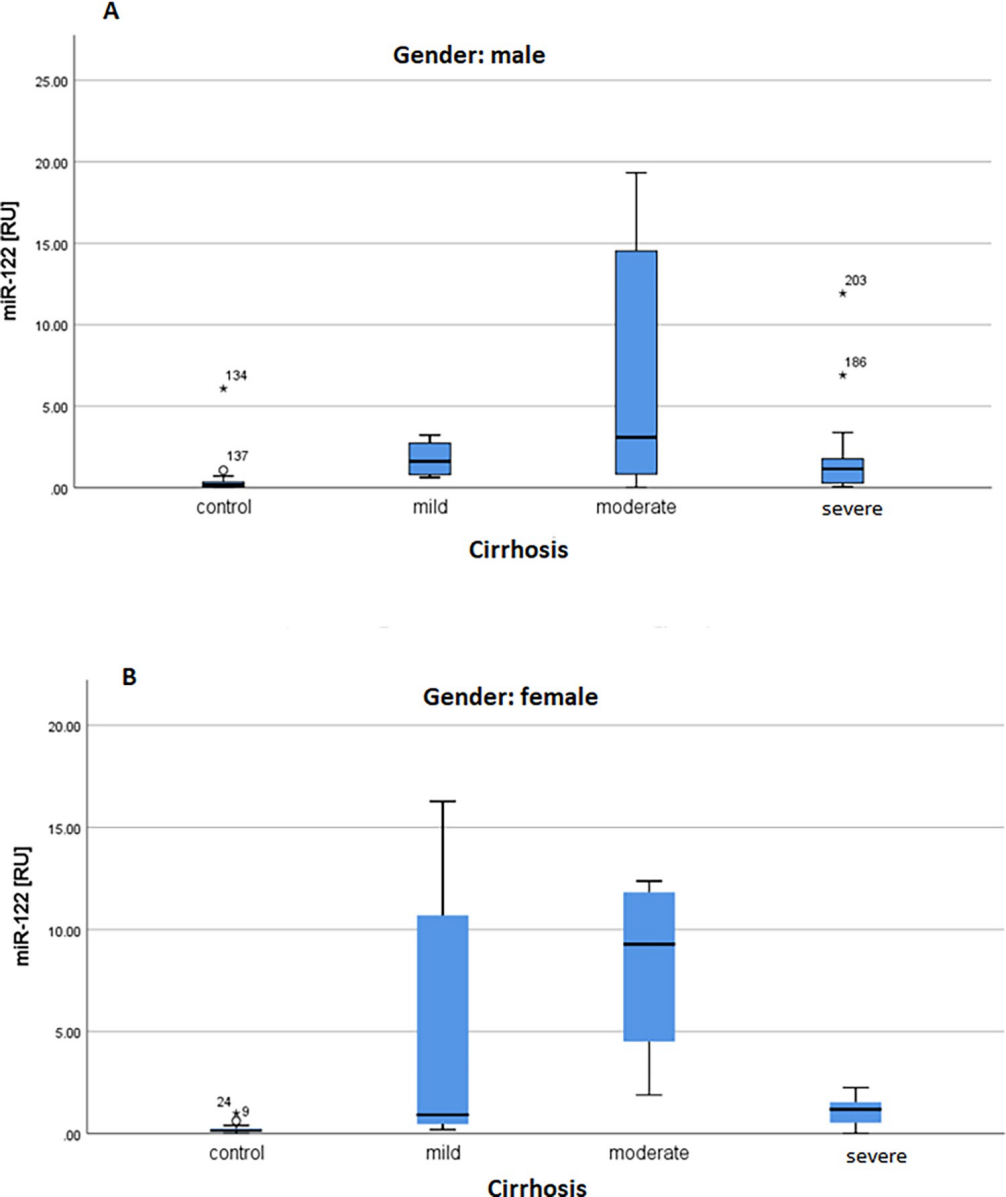
**A, B:** Boxplot of microRNA-122 steps: in cirrhotic patients.

**Fig 7:**
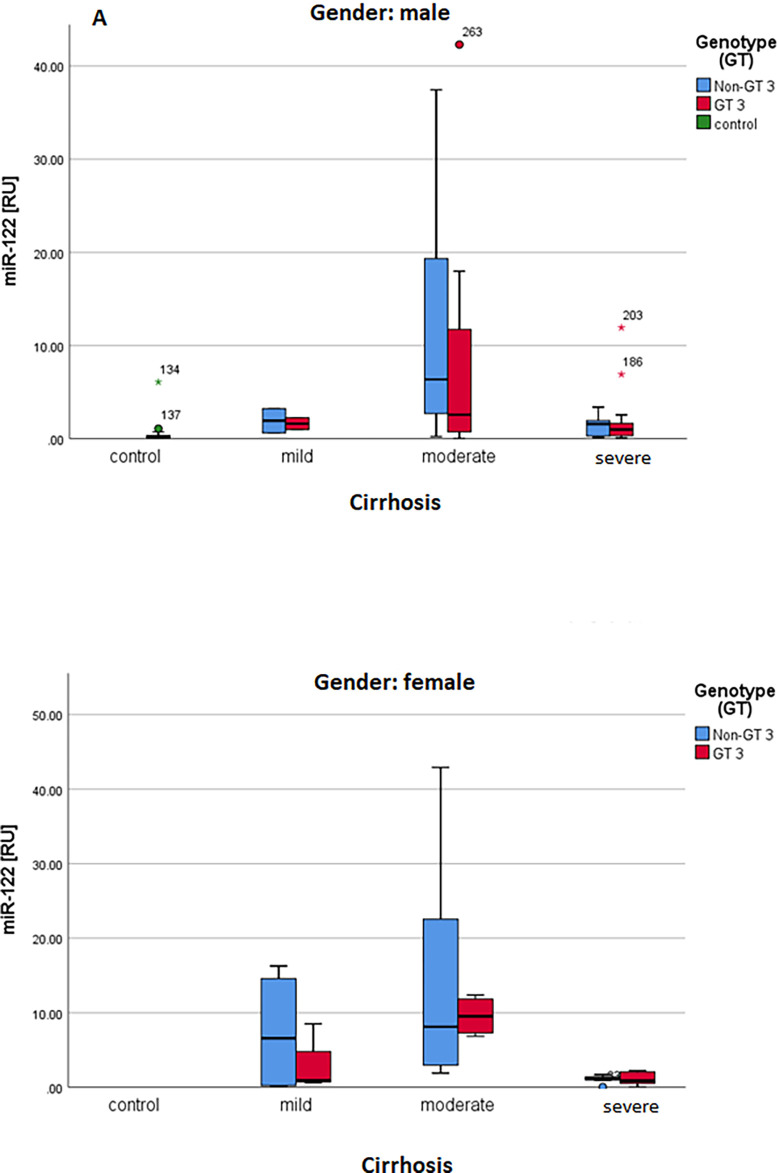
**A, B:** Grouped boxplot of microRNA-122 steps: cirrhosis and genotypes.

**Fig 8:**
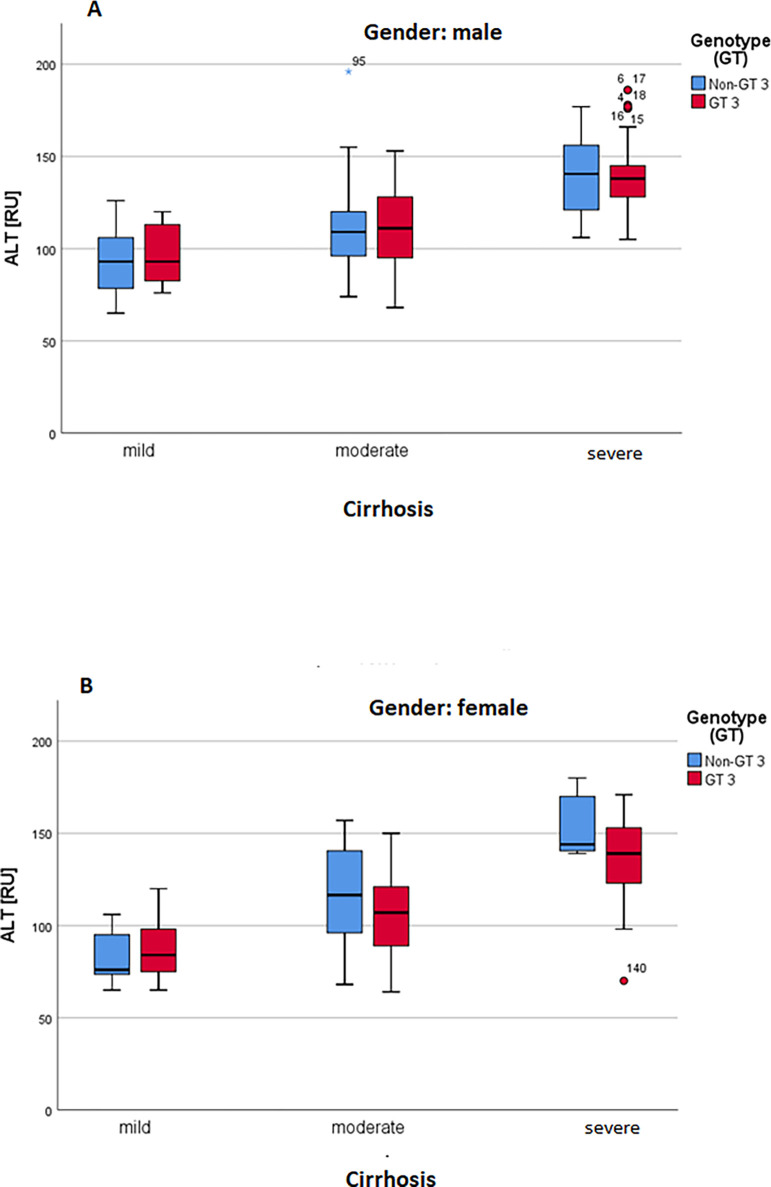
**A, B:** Grouped boxplot of ALT steps: cirrhosis and genotypes.

**Fig 9:**
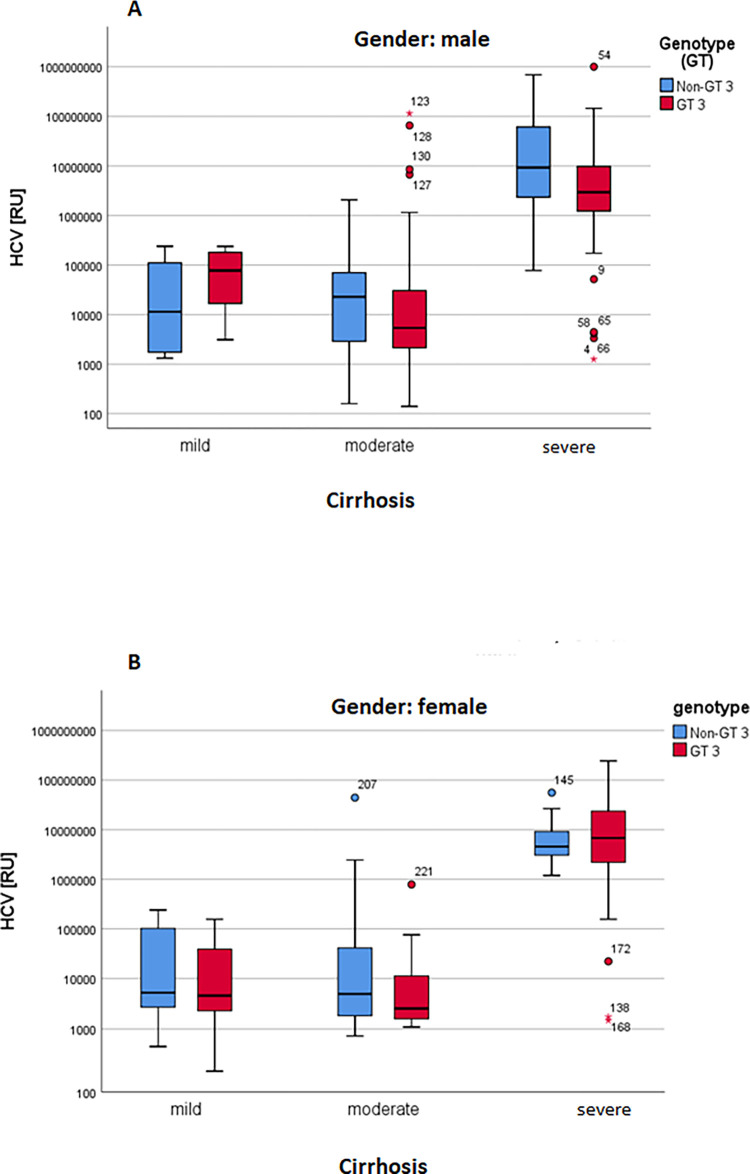
**A, B:** Grouped boxplot of viral load steps: cirrhosis and genotypes.

MicroRNA-122 was significant upregulated in the male and female group compared with healthy control as shown in the Fig 6A and 6B. Similary, in both male and female the microRNA was expressed higly in the genotype 3 pattient as compared non genotype with control group (Fig 7A and 7B). Based on the ALT, the miR-122 in male and female significantly expressed in the liver as shown in the Fig 8A and 8B. In Fig 9A and 9B demonstrated viral load in genotype 3 and non genotype 3 patients of male and femal of HCV cirrhotic groups. This clearly revealed that due to severity of the disease viral load is increasing in the genotype 3 and non genotype 3 patients.

**Fig 10:**
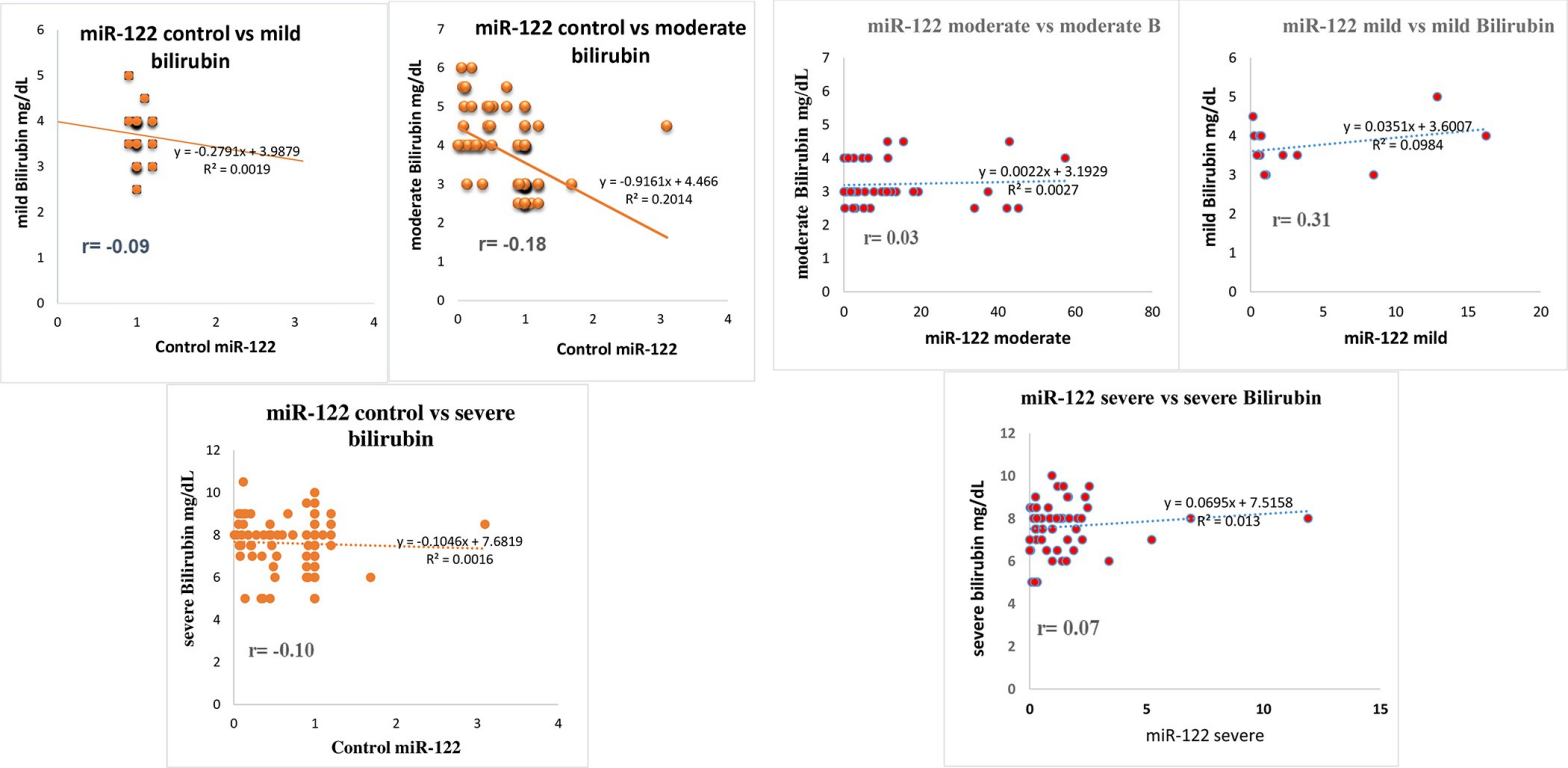
**A:** Correlation of bilirubin group with healthy control of miR-122. **B:** Correlation of bilirubin group with miR-122 HCV cirrhotic groups.

There was no correlation reported in the mild, moderate and severe bilirubin as compared to the healthy control of miR-122 reported in this study (S1 Table). Similarly, no correlation was found in the HCV group of mild, moderate and severe miR-122 comparing with mild, moderate and severe bilirubin as shown in the Fig 10A and 10B.

There was no Pearson’s correlation (r) reported in the mild, moderate and severe bilirubin as compared to the healthy control of miR-122 in this study. Similarly, no correlation was found in the HCV group of mild, moderate and severe miR-122 comparing with mild, moderate and severe bilirubin as shown in the Fig 10A and 10B.

## Discussion

MicroRNAs are becoming the accurate and non-invasive biomarkers of diagnosis for hepatic disease soon. Currently, there are no such diagnostic markers to detect liver diseases in their initial stages. The role of miRNAs in the regulation of HCV through modification of the host genes expression has been established recently [17, 24, 25]. In the present study, a total of 222 liver cirrhotic patients from the Khyber Pakhtunkhwa, Pakistan, were classified according to mild, moderate, and severe cirrhosis. Genotype 3 (a, b) is the predominant genotype recorded in Pakistan as in most Asian regions [20]. HCV genotype 3 was reported to occur particularly in patients with severe cirrhosis that have raised higher ALT levels as compared to patients with mild and moderate cirrhosis. This is in agreement with previous findings, describing more severe cirrhosis progression in patients with HCV genotype 3 infections [21].

As we and others have shown by previous studies [26–29], overall the miR-122 was highly expressed and significantly up-regulated (p = 0.0001) in HCV patients as compared to the healthy blood donors. Importantly, circulating miR-122 levels were highest in patients with beginning mild cirrhosis, whereas levels in serum samples from patients with more advanced cirrhosis were only moderately increased, confirming previous findings [26]. Hence, in accordance with previous data [21], our comparison of the miR-122 pattern between genotype 3 and non-genotype 3 HCV infected patients with mild, moderate, and severe cirrhosis proved that the pronounced increase of circulating miR-122 in the early inflammatory phase of fibrosis was also observed in the patient cohort, which was infected with HCV genotype 3. Serum miR-122 levels were assumed to be elevated at the stage of mild fibrosis due to high inflammatory activity and associated hepatocyte damage. Trebicka et al. [26], suggested that after the development of cirrhosis, miR-122 is released in low quantities because of the loss parenchymal tissue, the decreased miR-122 levels in damaged hepatocytes as well as the scarring barrier. But since unlike miR-122, the levels the serum ALT levels were not reduced with the severity of fibrosis, we hypothesize that the lower miR-122 levels in the blood after developing severe fibrosis are mainly due to the HCV-infected and damaged hepatocytes, which have lower miR-122 contents, leading then to a reduced release into the blood. Thus, the marked increase of serum miR-122 levels in the beginning stage of cirrhosis is not dependent on the genotype. Oliveira et al. also found no difference in the increase of circulating miR-122 levels between serum samples of genotype 1 and 3 patients, whereas in liver biopsies the miR-122 changes were more pronounced upon genotype 3 than genotype 1 HCV infection [30]. Since the genotypes 1 and 2 are rarely found in Pakistan, we did not report to the significance of miR-122 in these genotypes 1 and 2. Therefore is an urgent need to increase the hepatic sample size to completely identify the genotypes and their role of microRNAs in this region.

MicroRNA-122 levels in healthy controls were compared with the three groups using Pearson correlation. The severe patients revealed significant positive correlation (r = 0.46, p = 0.006). Interestingly, based on patient groups, the analysis of the miR-122 indicated that severe patients were sternly affected as compared to moderate and mild. Similarly, the patient groups were also evaluated and correlated with alanine amino-transaminase (ALT) levels. The severe patients had a high level of ALTs as compared to the moderate and mild groups. Besides this, the other two groups (mild and moderate) of miR-122 demonstrate a negligible correlation with ALT. This data is in agreement with the previous findings of Bihrer et al. who reported that the high levels of miR-122 in serum samples obtained from patients with chronic hepatitis C were associated with ALT activities, necro-inflammatory activity, and levels of ALT [24].

Although our study suffered from small sample size in cirrhosis, we performed at ROC analysis. The sensitivity rate of miR-122 was significant in the HCV cirrhotic patients. The study further describes that comparison of healthy control with mild patients’ area under the ROC curve (0.8476) was significant (p = 0.0001, 95% confidence interval 0.7359 to 0.9593) sensitivity rate as compared to moderate and severe group. There is no true positive rate reported between moderate and severe patients and hence no significant ROC and area AUC was found. The overall result of the miR-122 area under the ROC curve was significantly true positive. The above consequences reveal the clinical potential of the serum miRNA panel, with enhanced specificity and sensitivity in the HCV cirrhotic patients.

The study of biochemical parameters, AFP and ALT, were compared with serum miR-122 to determine the level of specificity and sensitivity. The AFP display no significant sensitivity results with miR-122 and the ALT was reported a significantly positive ROC curve with mild, moderate, and severe patients. Therefore, all the above outcomes of our work are closely related to the study of Weis et al., Butt et al., and El-Garem et al. [31–33].

The study has some of limitations as one of the main is sum of the cirrhotic samples were small and funding available for the project was less. Besides this, we did not reported the significance of microRNAs in genotypes 1 and 2 as in small quantity as these genotypes were rarely found in our country. There is urgently need to further study on a larger number of hepatic samples to completely identify the genotypes and their role of microRNAs of this region.

In conclusion, miR-122 has a vital role in HCV replication and significantly expressed in HCV cirrhotic patients as compared to healthy controls. Notably, our data demonstrate that the increased serum miR-122 levels, indicating sensitively hepatic disease progression, do not depend on the HCV genotype though genotype 3 HCV infection is known to be associated with more severe liver cirrhosis and steatosis.

## Supporting information

S1 Fig Expression of miR-122 in HCV cirrhotic patients. (TIF)

S1 Table Genotypes-comparison, correlation with C-b and correlations with C-Mir. (XLSX)

## List of abbreviations

AFP: alpha-fetoprotein
ALT: alanine transaminase
AST: aspartate aminotransferase
AUC: area under curve
HCC: hepatocellular carcinoma
HCV: hepatitis C virus, miR-122: microRNA-122
ROC: receiver operating characteristic
SPSS: statistical package for social science
SVR: sustained virological response
