# Supplementary figures and images for: Circulating microRNA-122 in HCV cirrhotic patients with high frequency of genotype 3
Source: PLoS One. 2022 May 26;17(5):e0268526. doi: 10.1371/journal.pone.0268526 (PMC9135289; doi:10.1371/journal.pone.0268526)

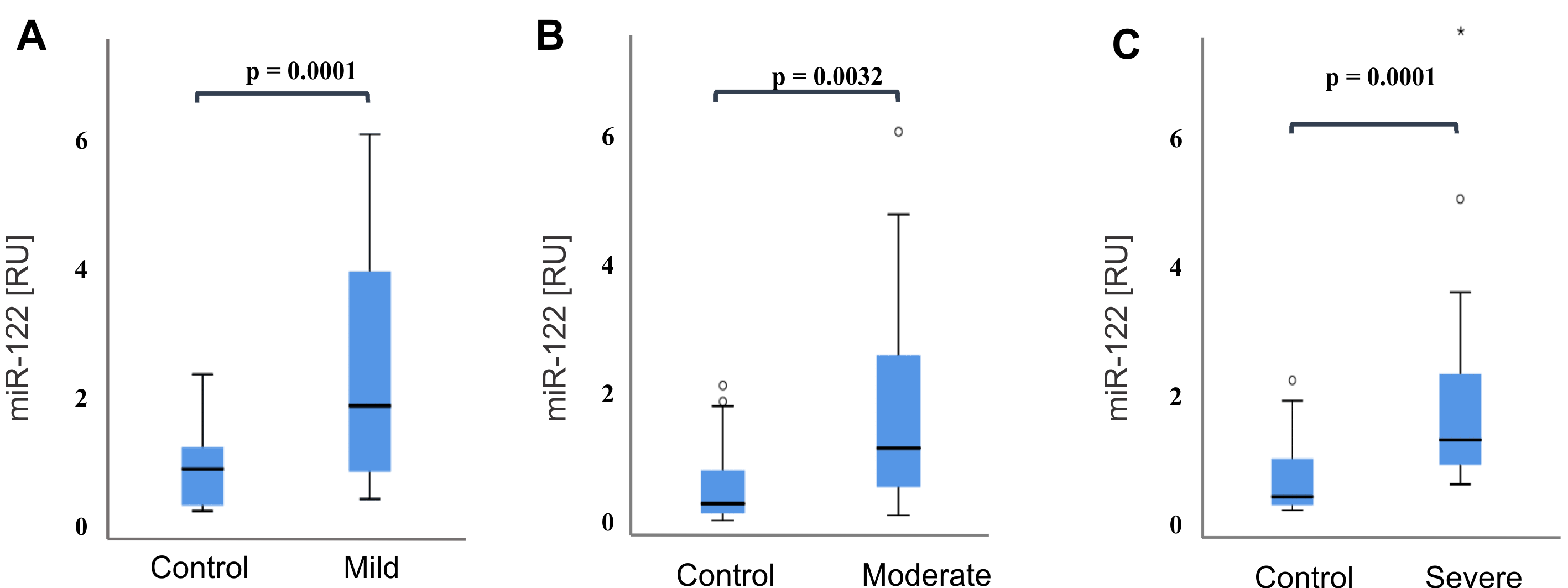

Supplement: S1 Fig — (TIF) [file pone.0268526.s001.tif]
